# Efficacy and safety of lumbrokinase plus aspirin versus aspirin alone for acute ischemic stroke (LUCENT): study protocol for a multicenter randomized controlled trial

**DOI:** 10.1186/s13063-022-06200-4

**Published:** 2022-04-11

**Authors:** Ying Chen, Yan Liu, Jingjing Zhang, Kehua Zhou, Xuecheng Zhang, Hengheng Dai, Baolin Yang, Hongcai Shang

**Affiliations:** 1 Key Laboratory of Chinese Internal Medicine of Ministry of Education, Dongzhimen Hospital, Beijing University of Chinese Medicine, No.5 Haiyuncang, Dongcheng District, Beijing, 100700 China; 2 Beijing University of Chinese Medicine, Beijing, 100029 China; 3 Department of Hospital Medicine, ThedaCare Regional Medical Center-Appleton, Appleton, WI 54911 USA; 4 Department of Encephalopathy, Dongzhimen Hospital, Beijing University of Chinese Medicine, Beijing, 100700 China

**Keywords:** Lumbrokinase, Aspirin, Acute ischemic stroke, Multicenter randomized controlled trial, Trial protocol

## Abstract

**Background:**

Lumbrokinase has been widely used for patients with acute ischemic stroke (AIS) in China; however, because rigorously designed studies are lacking, safety and efficacy of lumbrokinase in the treatment of acute ischemic stroke remains largely unknown. In this multicenter, randomized, and controlled trial, we aim to compare lumbrokinase plus aspirin versus aspirin alone in patients with acute ischemic stroke.

**Methods:**

A total of 220 eligible participants will be randomized to either the intervention or control group with a 1:1 ratio. These participants must be diagnosed with acute ischemic stroke for the first time, whose symptoms appear within 72 h. Their NIHSS score must be greater than 5 and less than 15, and their age must be between 35 and 85 years old. They must have not received intravenous thrombolysis, arterial thrombolysis, or intravascular intervention. Participants in the intervention group will be treated with lumbrokinase plus aspirin for the first 90 days. Participants in the control group will use placebo plus aspirin for the first 90 days. Then, all participants will be treated with aspirin only and followed up for another 90 days (180-day follow-up). The primary outcome is the modified Rankin Scale (mRS) score. The secondary outcomes are National Institutes of Health Stroke Scale (NIHSS) score, Activity of Daily Living (ADL) Scale score, coagulation function, and serum hypersensitive C-reactive protein. The exploratory outcomes are fasting lipid panel, recurrence rate, the occurrence of cardiovascular and cerebrovascular events, and the mortality rate. Safety evaluations include liver function and kidney function, serum fibrinogen, adverse events, serious adverse events, and bleeding events. Adherence of participants will also be assessed.

**Discussion:**

This trial will investigate the efficacy and safety of lumbrokinase plus aspirin as compared to aspirin alone in the treatment of acute ischemic stroke.

**Trial registration:**

Chinese Clinical Trial Registry, ChiCTR2000032952. Registered on May 16, 2020.

## Administrative information

Note: the numbers in curly brackets in this protocol refer to SPIRIT checklist item numbers. The order of the items has been modified to group similar items (see http://www.equator-network.org/reporting-guidelines/spirit-2013-statement-defining-standard-protocol-items-for-clinical-trials/).

**Table:** 

Title {1}	Efficacy and safety of lumbrokinase plus aspirin versus aspirin alone for acute ischemic stroke (LUCENT): study protocol for a multicenter randomized controlled trial
Trial registration {2a and 2b}.	Chinese Clinical Trial Registry, which is the primary registry of the WHO International Clinical Trial Registration Platform, ChiCTR2000032952. Registered on May 16, 2020. URL: http://www.chictr.org.cn/index.aspx
Protocol version {3}	Version 2.0 of 28-12-2019
Funding {4}	National Science Fund for Distinguished Young Scholars, China (No.81725024)
Author details {5a}	Ying Chen: Key Laboratory of Chinese Internal Medicine of Ministry of Education, Dongzhimen Hospital, Beijing University of Chinese Medicine, Beijing 100700, China; Beijing University of Chinese Medicine, Beijing 100029, China Yan Liu: Key Laboratory of Chinese Internal Medicine of Ministry of Education, Dongzhimen Hospital, Beijing University of Chinese Medicine, Beijing 100700, China Jingjing Zhang: Key Laboratory of Chinese Internal Medicine of Ministry of Education, Dongzhimen Hospital, Beijing University of Chinese Medicine, Beijing 100700, China; Beijing University of Chinese Medicine, Beijing 100029, China Kehua Zhou: Department of Hospital Medicine, ThedaCare Regional Medical Center-Appleton, Appleton, WI 54911, USA Xuecheng Zhang: Key Laboratory of Chinese Internal Medicine of Ministry of Education, Dongzhimen Hospital, Beijing University of Chinese Medicine, Beijing 100700, China; Beijing University of Chinese Medicine, Beijing 100029, China Hengheng Dai: Key Laboratory of Chinese Internal Medicine of Ministry of Education, Dongzhimen Hospital, Beijing University of Chinese Medicine, Beijing 100700, China; Beijing University of Chinese Medicine, Beijing 100029, China Baolin Yang: Department of Encephalopathy, Dongzhimen Hospital, Beijing University of Chinese Medicine, Beijing 100700, China Hongcai Shang: Key Laboratory of Chinese Internal Medicine of Ministry of Education, Dongzhimen Hospital, Beijing University of Chinese Medicine, Beijing 100700, China
Name and contact information for the trial sponsor {5b}	Investigator initiated clinical trial;Hongcai Shang (Principal Investigator)shanghongcai@126.com
Role of sponsor {5c}	This is an investigator initiated clinical trial. Therefore, the funders played no role in the design of the study and collection, analysis, and interpretation of data and in writing the manuscript.

## Introduction

### Background and rationale {6a}

Stroke is a major cause of mortality and morbidity worldwide. Each year, 15 million people are diagnosed with stroke globally; of them, 5 million suffer from death and another 5 million become permanently disabled [1]. Ischemic stroke accounts for approximately 80 to 87% of all strokes [2]. Among patients with a minor ischemic stroke or a transient ischemic attack (TIA), 3 to 15% will get ischemic stroke 90 days later [3]. As a traditional and the most widely used antiplatelet therapy, aspirin reduces the risk of early recurrent ischemic stroke and improves long-term outcomes without a risk of hemorrhagic complications. However, breakthrough ischemic strokes are not uncommon in patients who are already taking aspirin monotherapy and an addition of a second antiplatelet therapy like clopidogrel significantly increases the risk of bleeding.

**Fig. 1:**
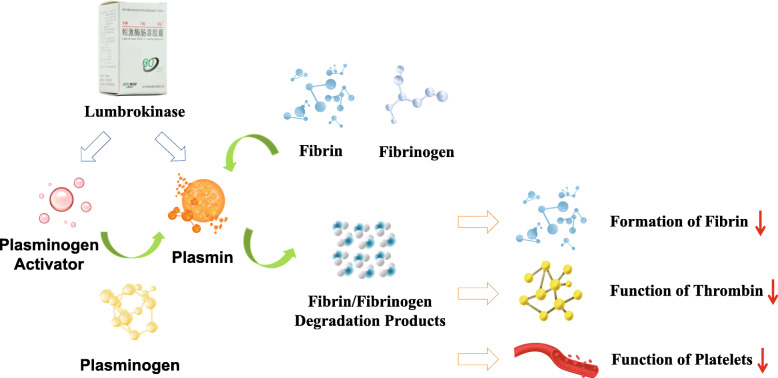
How lumbrokinase works

In the current Chinese guidelines for the treatment of acute ischemic stroke (AIS), lumbrokinase is listed as a defibrinogenating agent with strong recommendation [4]. Lumbrokinase is extracted from special earthworms (*Lumbricus rubellus*) [5]. It is actually a name of a group of bioactive proteolytic enzymes including plasminogen activator and plasmin [6]. Unlike tissue plasminogen activator (t-PA), lumbrokinase does not have the side effects of excessive bleeding or heavy blood loss, although it converts plasminogen to plasmin and dissolves fibrin clots (Fig. 1) [7]. Lumbrokinase reduces fibrinogen, prolongs prothrombin time (PT) and activated partial thromboplastin time (APTT), reduces plasma viscosity, and inhibits platelet aggregation. In a meta-analysis of 4751 patients from 33 randomized controlled studies, You et al. found that lumbrokinase could facilitate the recovery of neurological functions and improve the activity of daily living, with mild side effects including nausea and vomiting, black stool (but stable hemoglobin levels), dizziness and other symptoms at a low frequency [8].

A number of randomized clinical trials also investigated the effects of lumbrokinase plus aspirin for AIS. Zuo found that lumbrokinase plus aspirin decreased plasma fibrinogen concentration, whole blood viscosity and plasma viscosity. Patients treated with lumbrokinase plus aspirin had lower NIHSS scores 12 months later [9]. Similarly, Zhou et al. found that patients treated with lumbrokinase plus aspirin had better outcomes in quality of life per Bathel index and Chinese Stroke Scale (CSS). Nonetheless, the available studies were usually of poor quality with various methodological limitations. Thus, we aim to investigate the efficacy and safety of lumbrokinase plus aspirin as compared to aspirin alone in the treatment of AIS.

### Objectives {7}

We aim to investigate the efficacy and safety of lumbrokinase plus aspirin as compared to aspirin alone in the treatment of AIS.

The primary outcome is the Modified Rankin Scale (mRS) score.

The secondary outcomes are National Institutes of Health Stroke Scale (NIHSS) score, Activity of Daily Living (ADL) Scale score (Barthel index), coagulation function: prothrombin time (PT), activated partial thromboplastin time (APTT), serum fibrinogen (FIB) concentration, and serum hypersensitive C-reactive protein (hs-CRP).

### Trial design {8}

**Fig. 2:**
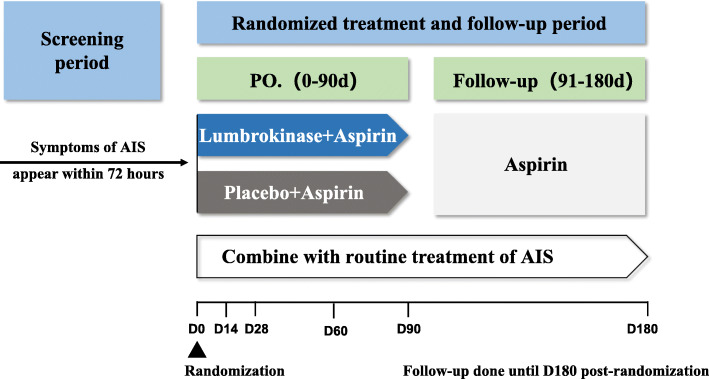
The flow diagram of this trial. PO, per os; AIS, acute ischemic stroke

In this prospective, multicenter, randomized, double-blind, placebo-controlled clinical trial, lumbrokinase plus aspirin is compared to aspirin alone. We plan to enroll 220 participants with first time diagnosis of AIS. The participant allocation ratio is 1:1. Participants will be treated for the first 90 days. Then, all participants will be treated with aspirin only and followed up for another 90 days (180-day follow-up). A flowchart of the study is shown in Fig. 2. Central ethical approval of LUCENT has been confirmed by the Ethics Committee of Dongzhimen Hospital, Beijing University of Chinese Medicine (No.DZMEC-KY-2019-202). This trial will be conducted in adherence to the Declaration of Helsinki. Informed consent will be obtained from all participants or their legal representative, in writing, before inclusion in the trial. The trial has been registered in the Chinese Clinical Trial Registry (ChiCTR2000032952).

## Methods: participants, interventions, and outcomes

### Study setting {9}

LUCENT will be conducted in ten hospitals across China from June 2020 to April 2022. They are Dongzhimen Hospital, Beijing University of Chinese Medicine, Dongfang Hospital, Beijing University of Chinese Medicine, Beijing University of Chinese Medicine Third Affiliated Hospital, Xuanwu Hospital Affiliated to Capital Medical University, the First Teaching Hospital of Tianjin University of TCM, Second Affiliated Hospital of Tianjin University of TCM, Tianjin Huanhu Hospital, Affiliated Hospital of Jiangxi University of Traditional Chinese Medicine, the First Affiliated Hospital of Nanchang University, the Second Affiliated Hospital of Nanchang University.

### Eligibility criteria {10}

The diagnosis of AIS in this trial is based on the guidelines from the Chinese Society of Neurology [10]. Participants are considered for inclusion if they meet the criteria as defined below.

1. Participant is diagnosed with acute ischemic stroke for the first time
2. Symptoms of acute ischemic stroke appear within 72 h
3. Before the onset of symptoms of acute ischemic stroke, the mRS score of the participant is less than or equal to 2
4. Participant has not received intravenous thrombolysis, arterial thrombolysis, or intravascular intervention
5. The participant’s NIHSS score is greater than 5 and less than 15
6. The age of the participant is between 35 and 85 years old
7. Agree to participate in the trial and signed informed consent form

Exclusion criteria are as follows:

1. The participant has a hemorrhagic cerebrovascular disease (including subarachnoid hemorrhage, cerebral hemorrhage, and other intracranial hemorrhage)
2. Transient ischaemic attack (TIA) and silent cerebral infarction
3. Known or suspicion for cardiogenic embolism: atrial fibrillation, dilated cardiomyopathy, infective endocarditis, mitral valve prolapse, sick sinus syndrome, atrial myxoma, patent foramen ovale, atrial septal defect, ventricular wall aneurysm, etc.
4. Carotid artery stenosis ≥70%
5. Blood pressure exceeds 180/100 mmHg (24/13.3 kPa) after active drug treatment
6. Blood homocysteine exceeds 20 μ mol/L after active drug treatment
7. Used defibrase within 24 h (including urokinase, snake venom preparation, batroxobin, lumbrokinase, etc.)
8. Have the following serious complications:

- Cardiac insufficiency: myocardial infarction, acute heart failure
- Hepatic insufficiency: total bilirubin>3 mg/dL, albumin<3 g/dL, ALT or AST increased to 3 times or more than the normal upper limit (≥120 IU/mL)
- Renal insufficiency: creatinine>178 μmol/L (2 mg/dL), urea nitrogen>18 mmol/L (40 mg/dL)

(9) Participant with bleeding tendencies (active gastric ulcers or bleeding) or hemorrhagic diseases or severe bleeding within 3 months, such as hemoptysis, hematochezia (fecal occult blood test), thrombocytopenic purpura, coagulopathy with INR > 1.7, active tuberculosis, etc., including platelet count<100 × 10^9^/L
(10) Participant has already taken anticoagulants or antiplatelet drugs or nonsteroidal anti-inflammatory drugs within 7 days prior to the stroke event
(11) Participant is allergic or intolerant to lumbrokinase or aspirin
(12) Participant with dysphagia and could not be given drugs orally
(13) Participant with a history of malignant tumor or aneurysm (including intracranial aneurysm or peripheral aneurysm)
(14) Women with recent pregnancy plans, as well as pregnant and lactating women
(15) Participant who had a surgery in the last 7 days
(16) Participant has a major mental illness and could not control his/her own actions, unable to cooperate with the trial
(17) Participant who is unable to understand the informed consent form or is unwilling to provide personal information or does not want to enter the trial
(18) Participant who participated in another clinical trial in the last 30 days

### Who will take informed consent? {26a}

Patients with AIS will be screened for eligibility to participate in this trial based on the abovementioned criteria. After the patient has been assessed as eligible by the treating physician, he/she will receive initial study information. Within 72 h, patients are invited to meet with the research physician to discuss any remaining questions and sign the informed consent.

### Additional consent provisions for collection and use of participant data and biological specimens {26b}

The collection and use of participants’ data will be stated in the informed consent. We will not collect biological specimens. There will be no additional consent provisions.

## Interventions

### Intervention description {11a}

Participants in both groups will receive standard medical care including aspirin 100 mg daily throughout the trial. Besides, participants in the intervention group will be treated with oral lumbrokinase (600,000 units), three times a day, half an hour before each meal plus oral aspirin 100 mg, once a day. Participants in the control group will be treated with placebo plus aspirin at a similar schedule. The course of treatment is 90 days with a follow-up period till day 180; after 90 days, all participants will continue the same aspirin treatment. An overview of data collection schedule is shown in Table 1. Additional medication use, adverse events (AEs), and serious adverse events (SAEs) will be recorded throughout the trial.

**Table 1:** Outcome evaluation time points

Time	Screening period	Baseline stage	Period of drug treatment(90d)				Follow-up
	− 72 h	D0	D14 ± 3 days	D28 ± 3 days	D60 ± 3 days	D90 ± 3 days	D180 ± 7 days
Visit date	X	X	X	X	X	X	X
Inclusion/exclusion criteria	X						
Informed consent	X						
Demographic data	X						
Vital signs and physical examination	X	X	X	X	X	X	
History of malignant tumor and aneurysm	X						
Family history, medical history and treatment history of TIA, Stroke, hemorrhagic disease	X						
Previous history of disease	X						
Allergic history	X						
History of tobacco and alcohol		X					
**Routine blood**							
RBC		X				X	
WBC		X				X	
Neutrophil		X				X	
Lymphocyte		X				X	
PLT		X				X	
Hb		X				X	
PCV		X				X	
**Urinalysis**							
U-LEU		X	X	X	X	X	
U-BLD		X	X	X	X	X	
R-PRO		X	X	X	X	X	
U-Ket		X	X	X	X	X	
**Routine stool**							
OB	X					X	
**Liver function and kidney function**							
ALT	X		X	X	X	X	
AST	X		X	X	X	X	
TBil	X		X	X	X	X	
BUN	X		X	X	X	X	
Scr	X		X	X	X	X	
**Fasting lipid panel**							
TC		X				X	
TG		X				X	
HDL		X				X	
LDL		X				X	
Fasting blood glucose		X				X	
**Coagulation function**							
PT		X	X	X	X	X	
APTT		X	X	X	X	X	
FIB		X	X	X	X	X	
hs-CRP		X				X	
Homocysteine	X						
ECG	X					X	
MRI of brain	X					X	
ADL scale (BI)		X				X	X
NIHSS	X		X	X	X	X	
mRS before stroke	X						
mRS		X	X	X	X	X	X
Recurrence rate			X	X	X	X	X
Cardiovascular and cerebrovascular events			X	X	X	X	X
Bleeding event			X	X	X	X	
Mortality rate			X	X	X	X	X
AE and SAE		X	X	X	X	X	
Combined use of drugs		X	X	X	X	X	
Adherence evaluation			X	X	X	X	

Abbreviations: *TIA*, transient ischaemic attack; *RBC*, erythrocyte count; *WBC*, leukocyte count; *PLT*, blood platelet; *Hb*, hemoglobin; *PCV*, packed cell volume; *U-LEU*, urine leukocyte; *U-BLD*, urine latent blood; *R-PRO*, urine protein; *U-Ket*, urine ketone bodies; *OB*, fecal occult blood; *ALT*, alanine aminotransferase; *AST*, aspartate aminotransferase; *TBil*, total bilirubin; *BUN*, blood urea nitrogen; *Scr*, serum creatinine; *TC*, total cholesterol; *TG*, triglyceride; *HDL*, high-density lipoprotein; *LDL*, low-density lipoprotein; *PT*, prothrombin time; *APTT*, activated partial thromboplastin time; *FIB*, fibrinogen; *hs-CRP*, hypersensitive C-reactive protein; *ECG*, electrocardiogram; *MRI*, magnetic resonance imaging; *ADL*, activity of daily living; *BI*, Barthel Index; *NIHSS*, National Institutes of Health Stroke Scale; *mRS*, Modified Rankin Scale; *AE*, adverse event; *SAE*, serious adverse event

### Explanation for the choice of comparators {6b}

For patients with acute ischemic stroke who do not conform to the indications of intravenous thrombolysis or intravascular thrombectomy, and have no contraindications, oral aspirin should be given as soon as possible after onset in China [4]. Aspirin can be changed to a prophylactic dose after the acute phase. So its selection as comparator is justified. In this trial, aspirin will be taken 100 mg once a day for 180 days. Its dose, frequency, and days are in accordance with the current practice in China. The side effects of aspirin in the drug instruction include the following: (1) Upper and lower gastrointestinal discomfort, such as indigestion, gastrointestinal, and abdominal pain; rash, urticaria, edema, and pruritus; cardiovascular and respiratory system discomfort. (2) Rare cases included gastrointestinal inflammation and gastroduodenal ulcers. (3) Very rarely, there may be gastrointestinal bleeding and perforation, hematoma, epistaxis, urinary genital bleeding, and gingival bleeding. (4) Particularly rarely, gastrointestinal bleeding, intracerebral hemorrhage, anaphylactic shock, and transient liver damage with elevated liver transaminase. (5) Dizziness and tinnitus were reported during drug overdoses. Nevertheless, the benefits of aspirin in patients with ischemic stroke outweigh the side effects, which is internationally recognized. In this trial, participants’ adverse events will be closely observed and treated in time.

### Criteria for discontinuing or modifying allocated interventions {11b}

Participants can leave the trial at any time for any reason if they wish to do so without any consequences. The participant’s participation in this trial can also be ended by the investigator if the participant is uncooperative and/or does not attend study visits. The participant’s data that have been collected up to that moment will be included in the analysis. This trial will be prematurely ended in case of any abundance in adverse events or procedure/compound-related complications or if the independent physician advises this termination. Criteria for trial termination include any suspected unexpected serious adverse event (SAE) based on an allergic reaction and clear allergic or iatrogenic effects in two or more participants.

### Strategies to improve adherence to interventions {11c}

In this trial, improving adherence to interventions will be through face-to-face reminders at the initial study drug dispensing and each follow-up thereafter. The reminders to participants include the following: (1) the importance of following study guidelines for adherence to take study drugs; (2) instructions about taking study drugs including dose timing, storage, and importance of taking study drugs whole, and what to do in the event of a missed dose; (3) notification that there will be a study drug count at every follow-up visit. Participants will return the unused study drugs and bottle at each follow-up visit. At the same time, unused study drugs will be counted and recorded in the CRF by investigators; (4) importance of calling the investigators if experiencing problems possibly related to study drugs such as symptoms, lost study drugs.

### Relevant concomitant care permitted or prohibited during the trial {11d}

During the trial, the use of drugs with the same effect as aspirin or lumbrokinase is prohibited.

### Provisions for post-trial care {30}

There is no ancillary or post-trial care. The trial has insurance, which is in accordance with the legal requirements in China. This insurance provides coverage for damage to participants through injury or death caused by any activities of the trial. The insurance applies to the damage that becomes apparent during the trial.

### Outcomes {12}

The primary outcome is the Modified Rankin Scale (mRS) score.

The secondary outcomes are National Institutes of Health Stroke Scale (NIHSS) score, Activity of Daily Living (ADL) Scale score (Barthel index), coagulation function: prothrombin time (PT), activated partial thromboplastin time (APTT), serum fibrinogen (FIB) concentration, and serum hypersensitive C-reactive protein (hs-CRP).

The safety outcomes are liver function and kidney function: alanine aminotransferase (ALT), aspartate aminotransferase (AST), total bilirubin (TBil), blood urea nitrogen (BUN), serum creatinine (Scr), and urine protein (R-PRO), as well as serum fibrinogen (FIB), occurrence of adverse events (AEs) and serious adverse events (SAEs), and occurrence of bleeding events. Adherence of participants will also be assessed.

The exploratory outcomes include fasting lipid panel: total cholesterol (TC), triglyceride (TG), high-density lipoprotein (HDL), low-density lipoprotein (LDL), and recurrence rate (incidence of ischemic stroke events), occurrence of cardiovascular and cerebrovascular events, and mortality rate. A study schedule and evaluation of outcomes are provided in Table 1.

The five elements of the primary outcome and secondary outcomes are specified in Table 2: the domain, specific measurement, specific metric, method of aggregation, and time point.

**Table 2:** Five elements of outcomes

	Domain	Specific measurement	Specific metric	Method of aggregation	Time point
Primary outcome	The Modified Rankin Scale (mRS) score	The Modified Rankin Scale (mRS) score	Change from baseline to 14, 28, 60, 90, and 180 days	Proportional-odds logistic-regression model	Baseline, 14, 28, 60, 90, and 180 days
Secondary outcome	National Institutes of Health Stroke Scale (NIHSS) score	National Institutes of Health Stroke Scale (NIHSS) score	Change from screening period to 14, 28, 60, and 90 days	Repeated-measures analysis model	Screening period, 14, 28, 60, and 90 days
	Activity of Daily Living (ADL) Scale score (Barthel index)	Activity of Daily Living (ADL) Scale score (Barthel index)	Change from baseline to 90 and 180 days	Repeated-measures analysis model	Baseline, 90 and 180 days
	Prothrombin time (PT)	Prothrombin time (PT)	Change from baseline to 14, 28, 60, and 90 days	Repeated-measures analysis model	Baseline, 14, 28, 60, and 90 days
	Activated partial thromboplastin time (APTT)	Activated partial thromboplastin time (APTT)	Change from baseline to 14, 28, 60, and 90 days	Repeated-measures analysis model	Baseline, 14, 28, 60, and 90 days
	Serum fibrinogen (FIB) concentration	Serum fibrinogen (FIB) concentration	Change from baseline to 14, 28, 60, and 90 days	Repeated-measures analysis model	Baseline, 14, 28, 60, and 90 days
	Serum hypersensitive C-reactive protein (hs-CRP)	Serum hypersensitive C-reactive protein (hs-CRP)	Change from baseline to 90 days	Repeated-measures analysis model	Baseline, 90 days

### Participant timeline {13}

Participant timeline is shown in Table 1.

### Sample size {14}

A sample size of 173 participants was calculated to provide 80% power to detect a common odds ratio (indicating the odds of improvement of 1 point on the mRS) of 6.02 in the distribution of scores on the mRS at 90 days between the treatment and control groups with a two-sided significance level of 0.05, assuming a similar between-group distribution of the mRS as in a recent AIS trial [11]. Taking into account of possible protocol violations and dropouts, we intend to enroll 220 participants (110 per group).

### Recruitment {15}

Participants will be recruited in ten hospitals across China. We will conduct recruitment in outpatient clinic, inpatient department, and emergency department. Participants will be recruited through four mechanisms: (1) suitable patients encountered by the investigators in their clinical work; (2) putting up posters in hospitals; (3) releasing relevant information through WeChat official account; (4) investigators regularly identify suitable participants in the hospitals’ medical imaging system.

## Assignment of interventions: allocation

### Sequence generation {16a}

Enrolled participants will be randomly assigned to the intervention group or the control group, allocated by the Interactive Web Response System (IWRS) with a ratio of 1:1. The randomization sequence will be generated in fixed block sizes and stratified by center. After informed consent, the investigators will log into IWRS to acquire a unique identification code and random number of each participant for participant identification and treatment assignment.

### Concealment mechanism {16b}

Allocation is concealed and will not be revealed to neither the participants nor the investigators upon randomization. Participants will be randomized by the Interactive Web Response System (IWRS), which is an online, central randomization service. IWRS will ensure the allocation concealment. It will only offer a random number to the investigator. Neither the investigators nor the participants will know from random numbers whether they represent the intervention group or the control group.

### Implementation {16c}

After signing the informed consent forms, the investigators will use the Interactive Web Response System (IWRS) to allocate the participant to one of the study arms.

## Assignment of interventions: Blinding

### Who will be blinded {17a}

Participants and investigators will be blinded. Blinding is ensured by using the double-blind technique. The lumbrokinase and the placebo of lumbrokinase will be identical in size, color, and smell. Each medication bottle will be labelled with a unique kit identity number, which corresponds to the random number obtained by IWRS. The number will be used to assign treatment without treatment allocation information. Outcome assessors or data analysts will also be blinded to treatment allocation. An employee outside the research team will export the data from the electronic CRF system so that outcome assessors or data analysts could analyze data without having access to the information about the allocation.

### Procedure for unblinding if needed {17b}

When a serious adverse event occurs, and it is difficult to judge the relationship between the event with interventions or overdose or serious drug interaction with the combined use of drugs, the investigator could reveal a participant’s allocated intervention in the IWRS.

## Data collection and management

### Plans for assessment and collection of outcomes {18a}

Investigators will be trained prior to recruiting participants and data will be managed as stated in Standard Operating Procedures (SOPs). Accuracy, reliability, and reference values of laboratory tests at each hospital will be unified prior to the start of this trial. Data from case report form (CRF) will be entered and stored in the electronic CRF system. A specialized quality monitor will regularly review CRFs and inspect the data. Monitoring results will be presented to the investigator-in-charge in each hospital, and the investigator-in-charge at each hospital is responsible for the accuracy, completeness, and timeliness of the recorded data. After all participants complete the 180-day visit and the data management team confirms all data have no issue, the database will be locked and then the outcome assessors or data analysts will begin to analyze the data under blinding. When data analysis is completed, the blinding will be open.

### Plans to promote participant retention and complete follow-up {18b}

The participants will receive extensive information about the study setup and requirements during the recruitment. The importance of completion of the follow-up will be stressed. Participants are allowed to stop at any time during the study and are not obliged to give a reason to discontinue. Throughout the follow-up period, the investigators will contact participants for completion of their follow-up.

### Data management {19}

In this trial, all data will be filled in the original case report form. Then they will be entered in the electronic case report form. Original case report forms will be kept on file at the participating site. Data integrity of the electronic case report form will be enforced through a variety of mechanisms, such as referential data rules, valid values, range checks, and consistency checks against data already stored in the database. Modifications to data entered in the electronic case report form will be documented through either the data change system or an inquiry system. An independent data monitoring committee (DMC) will periodically monitor the data entry of each hospital. The executive committee will publicize the enrollment of all hospitals on a weekly basis.

### Confidentiality {27}

Research data will be stored using a participant’s number for each participant. The key to the participant’s number will only be available to the research team during the study. No participant identification details will be reported in publications.

### Plans for collection, laboratory evaluation, and storage of biological specimens for genetic or molecular analysis in this trial/future use {33}

This trial will not collect biological specimens.

## Statistical methods

### Statistical methods for primary and secondary outcomes {20a}

The primary outcome will be analyzed by fitting a proportional-odds logistic-regression model to calculate the common odds ratio as a measure of the likelihood that the treatment group will lead to lower mRS scores than the control group (shift analysis).

For secondary outcomes, categorical variables will be compared with chi-square test or Fisher exact test. Longitudinal continuous variables will be analyzed by fitting a repeated-measures analysis model. Additionally, adverse events, serious adverse events, and laboratory abnormalities will also be compared using the chi-square test or Fisher exact test as appropriate.

All analyses will be performed in the intention-to-treat population using SAS version 9.4 (SAS Institute Inc). *P* values of less than 0.05 will be considered statistical significance, and all tests will be two-sided. No adjustments will be made for multiple comparisons.

### Interim analyses {21b}

There are no interim analyses planned.

### Methods for additional analyses (e.g., subgroup analyses) {20b}

There are no subgroup analyses planned.

### Methods in analysis to handle protocol non-adherence and any statistical methods to handle missing data {20c}

The primary outcome will be assessed using an intention-to-treat analysis. Missing data will be reduced to a minimum by using the appropriate measures described above. If any statistical method is needed to account for missing data in the secondary outcomes, multiple imputation will be used.

### Plans to give access to the full protocol, participant-level data, and statistical code {31c}

The data sets generated and/or analyzed during the current study are not publicly available, owing to the protection of privacy for participants, but they are available from the corresponding author on reasonable request.

## Oversight and monitoring

### Composition of the coordinating center and trial steering committee {5d}

*Principle investigator* takes supervision of the trial and medical responsibility of the participants.

*Data manager* organizes data capture and safeguards quality and data.

*Study coordinator* is responsible for the trial registration and coordinates study visits and annual safety reports.

*Study physician* identifies potential recruits, takes informed consent, and ensures follow-up according to protocol.

There is no trial steering committee or stakeholder and public involvement group.

### Composition of the data monitoring committee, its role and reporting structure {21a}

An independent data monitoring committee (DMC) will review on an ongoing basis accumulating study data to safeguard the interests of the participants. The DMC will assess the benefit/risk profile of the intervention during the study, ensure the validity and integrity of the study, review the overall conduct of the study, and provide recommendations to the Executive Committee (EC) regarding the continued conduct of the study [12].

### Adverse event reporting and harms {22}

All adverse events reported by the participants or observed by the investigators will be recorded. Adverse events will begin to be collected after the participant has provided consent and enrolled in the trial. An adverse event is recorded at any time. When an adverse event occurs, it is recorded immediately. If a participant experiences an adverse event after the informed consent document is signed (entry) but the participant has not started to receive any study intervention, the event will be reported as not related to the study drug [13]. All adverse events occurring after entry into the trial and until the 90th day will be recorded. An adverse event that meets the criteria for a serious adverse event (SAE) between study enrollment and the 90th day will be reported to both the ethics committee of the hospital, and the Ethics Committee of Dongzhimen Hospital, Beijing University of Chinese Medicine as an SAE. A serious adverse event for this trial is any untoward medical occurrence that is believed by the investigators to be causally related to study drug and results in any of the following: after receiving the study drug, the participants need to be hospitalized, need to prolong the length of hospital stay, become disabled, be life threatened or dead, have congenital malformation, and other events. Investigators will evaluate the causal relationship between adverse events and study drugs.

### Frequency and plans for auditing trial conduct {23}

Clinical research monitoring units will carry out regular monitoring. At the start of the trial, the monitors will conduct a tutorial on the electronic case report form. As the trial progresses, they will audit the overall quality and completeness of the data, examine source documents, interview investigators, and confirm that the hospital has complied with the requirements of the protocol. The monitors will verify that all adverse events are documented in the correct format and are consistent with protocol definition. Scheduling monitoring visits will be a function of patient enrollment, site status, and other commitments. They will notify the hospital at least 3 weeks prior to a scheduled visit. If a problem is identified during the visit, the monitor will assist the investigators in resolving the issues.

### Plans for communicating important protocol amendments to relevant parties (e.g., trial participants, ethical committees) {25}

All substantial amendments will be notified to ethical committees. In case amendments concern or affect participants in any way, they are informed about the changes. If needed, additional consent will be requested and registered.

### Dissemination plans {31a}

Results of this research will be disclosed completely in international peer-reviewed journals. Both positive and negative results will be reported.

## Discussion

Acute ischemic stroke most often presents as a minor stroke, and its risk of recurrent ischemic events is high, particularly in the first few days [12]. Aspirin is the only antiplatelet agent with a class 1A recommendation in international guidelines [14–16]. However, 15–25% of patients with a recent ischemic stroke who are being treated with aspirin suffer a new ischemic stroke. This phenomenon is known as aspirin resistance [17]. The failure of aspirin in preventing the recurrence of ischemic stroke is not only related to aspirin resistance, but may also be related to the increase in inflammatory mediators, like fibrinogen, which is a key player in atherogenesis, thrombogenesis, or ischemia distal to atherothrombotic stenoses or occlusions [18]. When the mean plasma concentration of fibrinogen is higher than normal 0.6 g/L, the risk of cardio-cerebral infarction or sudden death is 84%. When plasma fibrinogen is greater than 5 g/L, the risk of thrombosis is 4 times higher than normal. Fibrinogen is an independent risk factor for cardiovascular and cerebrovascular diseases [19]. William et al. studied the association between fibrinogen with recurrent vascular events after stroke. They prospectively recruited patients with acute stroke (*n* = 817) and followed them for up to 4 years for the occurrence of fatal or nonfatal recurrent stroke. They found the adjusted incidence of the outcome cluster recurrent stroke was significantly higher with higher levels of fibrinogen (75th to 25th percentile hazard ratio, 1.45; 95% CI, 1.24–1.72) [20].

Lumbrokinase can dissolve fibrinogen and fibrin [7]. Based on previous trials [9, 21], Lumbrokinase plus aspirin were more effective in reducing recurrence of ischemic stroke than aspirin alone and did not increase major bleeding events. As side effects of bleeding are more widely known with the available antiplatelet agents, like clopidogrel, lumbrokinase may open a new window for antiplatelet therapy with no increase in hemorrhagic events if proved. This will not only benefit patients with ischemic stroke, but also potentially patients with carotid stenosis and coronary artery disease who require the placement of drug eluting stents.

These hypotheses are based on the premise that the findings of the previous trials are true. However, the methodological deficiencies of these trials such as inadequate randomization, no double blinding, no placebo control, and incomplete outcome data might lead to various biases. Because of lacking robust evidence, efficacy of lumbrokinase plus aspirin for AIS remains to be established. A well-designed RCT is the gold standard for evaluating the clinical efficacy and safety of medicine [22]. LUCENT is a rigorously designed RCT to evaluate the efficacy and safety of lumbrokinase plus aspirin for AIS. In this trial, the IWRS and double-blind method will be used to avoid the potential selection and detection biases. Furthermore, in the LUCENT trial, patients will be treated for 90 days, followed by a follow-up period of 90 days. The relatively long follow-up period of 90 days will make it possible to assess the long-term effects of lumbrokinase plus aspirin.

There are also some limitations in this trial. We recruited only patients with mild or moderate ischemic stroke at the time of onset and lack of patients with severe ischemic stroke at the time of onset. Therefore, it is necessary to continue the trial of patients with severe ischemic stroke in future trials.

In this trial, we will be able to explore the potential role of lumbrokinase plus aspirin for reducing recurrence of ischemic stroke. The results of this trial will provide clinicians in China direct data in selecting therapeutic options for AIS and may also potentially open a new window for antiplatelet therapy in the cardiovascular and cerebrovascular medicine fields.

## Trial status

Recruiting started in September 2020. The current protocol is version 2 of 28-12-2019. Currently (29th of November 2021), we have recruited 46 participants. Participant recruitment is estimated to be completed around August 2022.

## Abbreviations

ADL: Activity of daily living
AE: Adverse event
AIS: Acute ischemic stroke
ALT: Alanine aminotransferase
APTT: Activated partial thromboplastin time
AST: Aspartate aminotransferase
BI: Barthel Index
BUN: Blood urea nitrogen
CRF: Case report form
CSS: Chinese Stroke Scale
DMC: Data monitoring committee
ECG: Electrocardiogram
EC: Executive committee
FIB: Fibrinogen
Hb: Hemoglobin
HDL: High-density lipoprotein
hs-CRP: Hypersensitive C-reactive protein
IWRS: Interactive Web Response System
LDL: Low-density lipoprotein
MRI: Magnetic resonance imaging
mRS: Modified Rankin Scale
NIHSS: National Institutes of Health Stroke Scale
OB: Fecal occult blood
PCV: Packed cell volume
PLT: Blood platelet
PO: Per os
PT: Prothrombin time
RBC: Erythrocyte count
RCT: Randomized controlled trial
R-PRO: Urine protein
SAE: Serious adverse event
Scr: Serum creatinine
SOP: Standardized operation process
TBil: Total bilirubin
TC: Total cholesterol
TG: Triglyceride
TIA: Transient ischemic attack
U-BLD: Urine latent blood
U-Ket: Urine ketone bodies
U-LEU: Urine leukocyte
WBC: Leukocyte count
